# A Method to Bond Vital Dentin With a Microleakage-Free Hybrid Layer

**DOI:** 10.3290/j.jad.c_1953

**Published:** 2025-04-03

**Authors:** Morakot Piemjai, Thanyarat Lerttriphob, Carat Jariyapayuklert, Franklin Garcia-Godoy

**Affiliations:** a Morakot Piemjai Professor, Department of Prosthodontics, Faculty of Dentistry, Chulalongkorn University, Bangkok, Thailand. Conceptualization, research design, project administration, supervision, methodology, formal analysis, data curation, validation, writing – original draft.; b Thanyarat Lerttriphob Graduate Student, Department of Prosthodontics, Faculty of Dentistry, Chulalongkorn University, Bangkok, Thailand. Methodology, sample preparation, investigation, data collection, and formal analysis.; c Carat Jariyapayuklert Master of Science Student, Department of Prosthodontics, Faculty of Dentistry, Chulalongkorn University, Bangkok, Thailand. Methodology, sample preparation, investigation, data collection, and formal analysis.; d Franklin Garcia-Godoy Professor, Department of Bioscience Research, College of Dentistry, University of Tennessee Health Science Center, Memphis, Tennessee, USA and Adjunct Faculty, The Forsyth Institute, Cambridge, Massachusetts, USA. Reviewing and editing the manuscript.

**Keywords:** intraoral microleakage-free restoration, MMA-TBB resin, primerless wet technique, 4-META/MMA-TBB resin, hybrid layer, tooth hypersensitivity

## Abstract

**Purpose:**

To investigate the dye penetration distance at Class V tooth-restoration margins/interfaces prepared in the laboratory and orally using a primerless wet technique and MMA-TBB bonding resin with or without 4-META monomer promoter.

**Materials and Methods:**

A box-form cavity at the cementoenamel junction was prepared on extracted human premolars and vital teeth scheduled for extraction for *in-vitro* and *in-vivo* studies, respectively. For each vital cavity, 1% citric acid and 1% ferric chloride aqueous (1-1) conditioner was applied for 10 s, 30 s, or 60 s, rinsed off and blot-dried, and was then bonded with either 4-META/MMA-TBB or MMA-TBB resin and bulk-filled with light-cured composite resin (n = 10). Restored vital teeth continued to function in the oral cavity for seven days before extraction. Restorations were stored in water at 37°C and 0.5% basic fuchsin dye solution for 24 h each before dye penetration measurement under a microscope, while a hybrid layer was observed using a scanning electron microscope (SEM).

**Results:**

No hypersensitivity or pain occurred in any vital teeth. For all 1-1 groups, no dye penetration was detected at any margins of the in- vitro restorations. Dye penetration (0.13 mm) was only observed in one intraoral restoration of 60 s etching with MMA-TBB resin at the cementum/dentin margin. A consistent hybrid layer after chemical modification was observed in leakage-free specimens.

**Conclusion:**

The results suggest that the 1–3 µm 1-1 demineralized substrate clinically provides sufficient permeability to form a microleakage-free hybrid layer using a primerless wet technique with MMA-TBB or 4-META/MMA-TBB resin. Intraoral microleakage-free restorations may lead to longer-term restored-tooth survival.

Clinically undetectable microleakage at the tooth-restoration margin/interface has long been unresolved, especially at cementum/dentin margins. Restorations with microleakage at the cementum/dentin margin/interface are vulnerable to postoperative hypersensitivity, marginal staining, secondary caries, restoration detachment, and pulpal pathology.^1,4,8^ Oral microorganisms or acids penetrate through this clinically undetectable leakage pathway, influencing the longevity of tooth vitality and restorations.^4^ Postoperative hypersensitivity of composite resin restorations is commonly found even with clinically acceptable performance, especially in posterior restorations.^3^ Oral stimuli increasing the movement of dentinal tubular fluid and pulpal sensory nerves are involved in tooth hypersensitivity.^2^ Therefore, preparing microleakage-free restorations to prevent oral stimuli is the best way to prevent these signs and symptoms.

Microleakage-free restorations can be made with a complete hybrid layer in the *in-vitro* studies.^18,21,25^ The hybridized tooth-resin interface without leakage or the complete hybrid layer resists the penetration of HCl and NaOCl solutions,^18,21,25^ or lactic acid, which is the primary cause of secondary caries in the oral cavity.^17^ The permeability and thickness of the demineralized tooth substrate and the ability of monomers to diffuse and impregnate are the key factors that form the hybrid layer.^9,14^ A molecular-level mixture of collagen and resin polymers formed in the subsurface of conditioned dentin is more complicated than enamel, even in the extracted teeth, because of the more organic nature of dentin.^9,14^ Dentin conditioned with phosphoric acid collapses with air drying, and it is difficult for monomers to diffuse entirely into it, which leads to severe leakage through the dentinal tubules and demineralized dentin at the margin/interface.^17,21,25^ Dentin conditioned for 10 s with 10-3 (10% citric acid and 3% ferric chloride aqueous mixture) and 4-META/MMA-TBB (4-methacryloyloxyethyl trimellitate anhydride/methyl methacrylate-tri-n-butylborane) resin (Super-Bond C&B, Sun Medical, Shiga, Japan) efficiently provide the leakage-free hybridized dentin in dry bonding.^17,18,21,25^ The scanning electron microscope (SEM) or transmission electron microscope (TEM) specimens prepared without epoxy embedding showed the detached or degraded dentin–resin interface or micro-gap formation in the tested leakage specimens using basic fuchsin, methylene blue, or silver nitrate tracers, while the non-detached consistent hybrid layer before and after chemical challenge was found in the leakage-free group.^18,21,25^ Restorations with dry or moist bonding techniques and phosphoric acid conditioning of dentin cannot provide a leakage-free resin-dentin interface.^6,17,21,25^

Prolonged 10-3 conditioning for 30–60 s and air-drying creates a demineralized dentin layer (>5 µm), too deep to be wholly impregnated by the auto-polymerized 4-META/MMA-TBB resin, resulting in a remaining demineralized dentin layer as a microleakage pathway.^18,25^ Meanwhile, the 1–3 μm complete hybrid layer without leakage using 1% ferric chloride in 1% citric acid aqueous (1-1) conditioning for 10–60 s and 4-META/MMA-TBB can be performed using wet bonding with primer^20^ or without primer (primerless wet technique: blot-dried without primer).^22–24^ The 1–3 μm 1-1 demineralized dentin provides easier dehydration and monomer impregnation; thus, the primer step is not required for completing the hybridization of 4META/MMA-TBB resin.^22^

Whether the 4-META in MMA-TBB resin is needed for complete hybridization when preparing the substrate surface with 1-1 conditioner in primerless wet condition is questionable. A former report shows that MMA-TBB resin has an inferior diffusion potential compared with 4-META/MMA-TBB resin. MMA-TBB resin does not bond to dried dentin even when conditioned with 10-3.^14^ However, bonding was possible if the monomer permeability of the dried demineralized dentin was improved by a nonionic surfactant^26^ or glutaraldehyde.^5^ Wet bonding with 4-META acetone primer and MMA-TBB resin showed cohesive failure in cured resin after tensile bond testing with a stable hybrid layer (4–5 μm) on dentin conditioned with ferric ion dissolved in 10% phosphoric acid.^7^ The presence of ferric chloride in the dentin conditioner helps decrease the amount of dissolved polyelectrolytes in the demineralized dentin by aggregating with ferric ions, which increases the permeability of demineralized dentin to water and acid.^19^ When blot-dried, the 1-1 demineralized dentin has higher permeability for monomer infiltration to form a complete hybrid layer than when it is air-dried.^24^ The next challenge is when the permeability of 1-1 demineralized dentin is sufficient so that MMA-TBB, without a monomer promoter such as 4-META, may give reliable dentin bonding without leakage.

However, the seal with leakage-free cementum/dentin margin and the interface is not easily accomplished in the vital tooth. The outward fluid flow from dentinal tubules and smears from tooth preparation are believed to have an adverse effect on the formation of a resin-reinforced layer *in vivo*.^10,15^ The hybrid layer (3–4 μm) prepared in the oral cavity by using 10 s 10-3 etching and 4-META/MMA-TBB resin in dry bonding demonstrates the resistance to the chemical challenge,^12^ and provides long-term survival of restored teeth with fewer complications.^16^ Therefore, a thinner hybrid layer of 1–3 μm would be more reliably performed *in vivo*. The study hypothesis was that the 1–3 µm depth of permeable demineralized substrate prepared with 1-1 conditioning for 10 s, 30 s, or 60 s and using a primerless wet technique could provide high permeability for MMA-TBB without 4-META monomer promoter to impregnate fully both *in-vitro* and *in-vivo* preparations.

The study’s purpose was to investigate the marginal seal of Class V composite resin restorations using primerless wet bonding when varying conditioning periods of 1-1 conditioner or 37% phosphoric acid and adhesives, MMA-TBB or 4-META/MMA-TBB resin, in the laboratory and in the mouth. Dye penetration was used to evaluate the microleakage pathway at the restoration margin and interface. The characteristics of tooth-resin interface before and after the chemical challenge was used to confirm the complete impregnation of monomers into the conditioned tooth substrate.

## MATERIALS AND METHODS

This study was approved by the Faculty Human Research Ethics Committee (approval numbers 048/2012 and 044/2018), Faculty of Dentistry, Chulalongkorn University). All methods were performed in accordance with the relevant guidelines and regulations. The experimental materials and methods were first investigated and analyzed *in vitro* before the clinical oral investigation *in vivo*.

### In-vitro Study

#### Preparation of restorations

Forty previously frozen human premolars extracted for orthodontic therapy were randomly allocated into four groups of 10 specimens each. A Class V box cavity of 3×3 mm and 1.5 mm depth was prepared with a diamond bur (204, Intensiv, Montagnola, Switzerland) on the buccal surface at the cementoenamel junction. The cavity preparation was prepared with a butt-joint cavosurface margin with one horizontal margin in enamel and the other in cementum. Cavity walls were either conditioned with 1-1 or 37% phosphoric acid (Bisco Co., IL, USA) for 10 s or 30 s and were then rinsed, and blot-dried for 10 s with absorbent paper (Kimtech Science, Kimwipes, New South Wales, Australia), and bonded with MMA-TBB and PMMA powder with a brush-dip technique (MMA from Mitsubishi Rayon Co., Tokyo, Japan; TBB catalyst and PMMA powder from Sun Medical Co., Shiga, Japan). All cavities were bulk-filled with composite resin (Metafil CX; Sun Medical Co., Shiga, Japan) and light-cured (Elipar TriLight, 3M ESPE, MN, USA) for 60 s. Fine diamond burs (Jota, Switzerland) in a high-speed handpiece were used to finish the restorations’ margins after being stored in water at 37ºC for 24 h.

#### Dye penetration measurement

All tooth surfaces, except for the restoration and 1 mm away from the horizontal occlusal (enamel) and gingival (cementum/dentin) margins, were coated twice with nail varnish (Ten Ten Nail Colour, Bangkok, Thailand) to standardize the dye penetration measurement. The root apices for all teeth were sealed with sticky wax. Whole tooth surfaces, except the root apex, were immersed in 0.5% basic fuchsin solution (Sigma-Aldrich, Saint Louis, USA) for 24 h. After cleaning with running tap water, each restored tooth was sectioned vertically through the midline of restoration with a diamond disk (270D, Intensiv, Montagnola, Switzerland) and a slow-speed handpiece. The two sectioned specimens were sequentially polished with silicon carbide grit paper No. 400, 600, 800, 1000, and 1200 (TOA, Samut Pragan, Thailand) with a polishing machine (Minitech 233, Presi, France). The margins and the distance of dye penetration on sectioned specimens were investigated under a microscope (Olympus, SZ61, Japan), and photographs were taken using an attached digital camera (Olympus, DP21, Japan) at ×10 to ×100 magnifications. Dye penetration distance was measured by one examiner using the Image Pro Plus program, and the highest leakage distance for each restoration was recorded for statistical analysis. The statistical significance in the mean leakage distance was set at α = 0.05.

#### Assessment of interfacial layer

Two teeth in each group were prepared and restored in the same manner as described previously for interfacial layer investigation under a SEM. The sectioned specimen (without epoxy embedding) surface was wet-polished from 600-grit to 1200-grit abrasive paper and 0.05 µm alumina paste, ultrasonically cleaned for 10 min. One polished section of each tooth was immersed in 6 mol/L hydrochloric acid (HCl) for 30 s, followed by 1 wt% sodium hypochlorite (NaOCl) for 60 min. After being gold-sputtered, the characteristics of the interfacial layer on the chemically treated and the polished specimens were examined under SEM at ×500 to ×10,000 magnifications.

The *in-vitro* results were analyzed first before the *in-vivo* study began. The methods that provided microleakage-free restorations and a consistent hybrid layer before and after the chemical challenge were carried out in an *in-vivo* study to follow the ethical guidelines.

### In-vivo Study

#### Intraoral preparation of restorations

Patients who had caries-free, vital, asymptomatic, but hopelessly periodontally-compromised teeth scheduled for extraction were included in the study. Patients with known allergies or intolerance to anesthesia or resin adhesives were excluded. The study procedures, available alternative treatments, and the risks involved were explained to the volunteer patients before written informed consent was obtained.

The vitality of all teeth was confirmed by using an electric pulp tester and the cavity test. Following local anesthesia (Scandonest 2% Special, Septodont, France) and rubber dam application, a Class V box cavity (3 mm × 3 mm × 1.5 mm) with butt-joint cavosurface enamel and cementum/dentin margins was prepared using a diamond bur (204, Intensiv, Montagnola, Switzerland) and a high-speed handpiece under air-water spray. Sixty cavities prepared on the buccal or lingual surfaces were randomly divided into six groups with 10 cavities each (n = 10) for three different etching periods and two bonding agents. The 1-1 conditioner was applied to the cavity walls either for 10 s, 30 s, or 60 s in each group. Each cavity was rinsed with water for 10 s, and the whole was blot-dried with absorbent paper and bonded with either 4-META/MMA-TBB resin (Sun Medical, Shiga, Japan) or MMA-TBB resin (MMA from Sigma-Aldrich, Saint Louis, USA and TBB from Sun Medical, Shiga, Japan) and PMMA powder (Sun Medical, Shiga, Japan) using a brush-dip technique. Composite resin (Metafil CX, Sun Medical, Shiga, Japan) was used to restore the cavities with a bulk-filled technique and light-cured (3M ESPE, MN, USA) for 30 s following a manufacturer’s instruction. The restorations were polished using fine diamond burs (5205L, Intensiv, Montagnola, Switzerland) and left under function in the oral cavity for 7 days. Before extraction, a dentist and the patients evaluated the signs and symptoms of the restored teeth. After extraction, using an atraumatic technique under local anesthesia, the restored teeth were cleaned under running tap water and stored in water at 37°C for 24 h.

#### Dye penetration measurement

All specimens were prepared for the dye penetration test as described earlier in the ‘Dye penetration measurement’ description.

#### Assessment of hybridized dentin

Two sectioned specimens of each restoration for dye penetration distance measurement in all groups were prepared for the SEM examination (without epoxy embedding) of polished and chemically challenged specimens. The sectioned surface was sequentially polished with abrasive papers from 600-grit to 1200-grit and 0.05 µm alumina paste, ultrasonically cleaned for 10 min, and air-dried. One polished section of each tooth was immersed in 6 mol/L HCl for 30 s, followed by 1 wt% NaOCl for 60 min. The characteristics of the hybrid layer were examined at ×500 to ×10000 magnifications.

## RESULTS

### In-vitro Study

**Fig 1:**
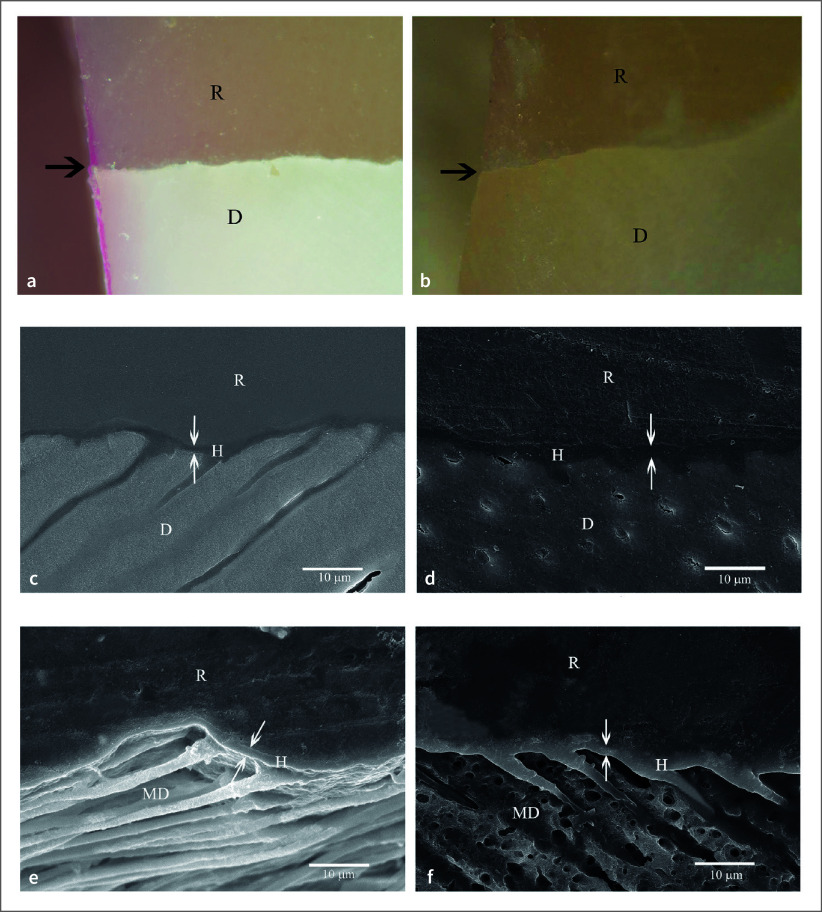
No dye penetration at cementum/dentin margin/interface (arrowed) of the *in vitro* MMA-TBB resin using 1-1 etched 10 s (a) and 30 s (b) (original ×100, D = dentin, R = restoration). The hybridized dentin layer was consistent after soaking in the HCl and NaOCl solutions: polished specimens of 10 s (c) and 30 s (d); chemically challenged specimens of 10 s (e) and 30 s (f) (original ×2000, D = dentin, MD = modified dentin, R = resin).

**Fig 2:**
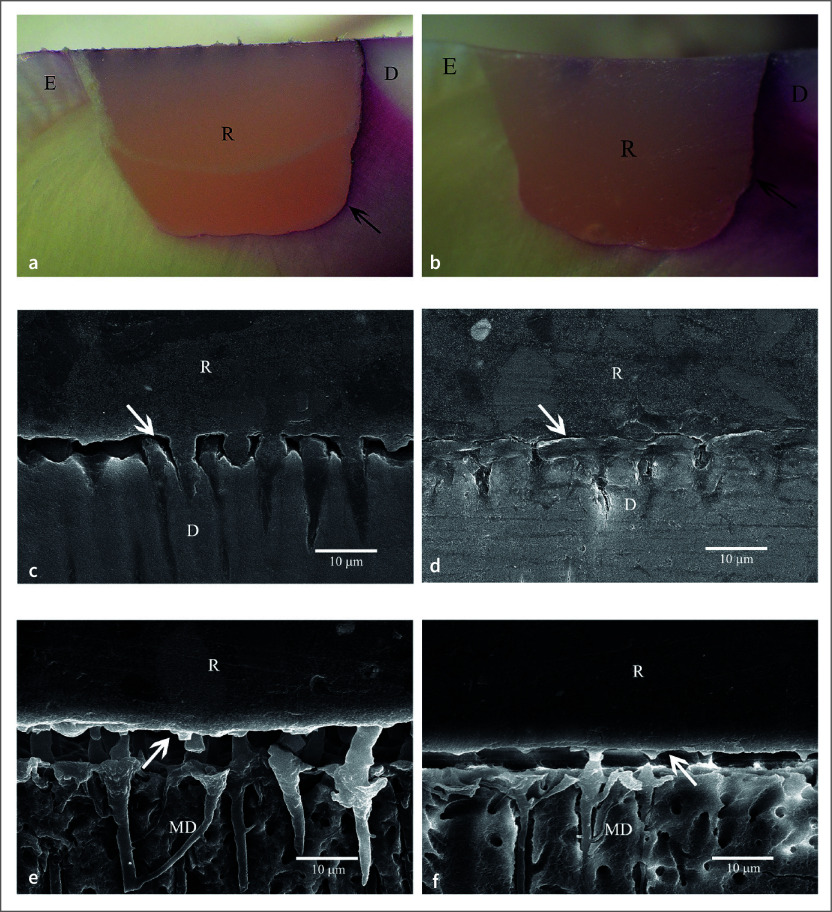
No dye penetration at enamel margin/interface but severe leakage at cementum/dentin margin/interface (arrowed) of the *in vitro* MMA-TBB resin using 37% phosphoric acid conditioning for 10 s (a) and 30 s (b) (original ×25, D = dentin, E = enamel, R = restoration). The detached dentin–resin interface (arrowed) in polished specimens of 10 s (c) and 30 s (d), and after soaking in the HCl and NaOCl solutions of 10 s (e) and 30 s (f) specimens. (original ×2000, D = dentin, MD = modified dentin, R = resin).

No dye penetration was found at the enamel-restoration interface in all groups and the cementum/dentin margin/interface in 1-1 etched 10 s and 30 s groups. Dye penetration distances at the cementum/dentin margin/interface between 10 s and 30 s phosphoric acid etched groups were not significantly different, analyzed using a Student’s t-test (p < 0.05) (Table 1). Leakage-free cementum/dentin margin/interface were observed in 1-1 etched 10 s and 30 s groups (Table 1, Figs 1a, 1b) with the 1–2 µm consistent hybrid layer resisting the chemical challenge (Figs 1c, 1d, 1e, 1f). The most severe leakage at the cementum/dentin-restoration interface was identified in phosphoric acid etched 10 s and 30 s groups (Table 1, Figs 2a, 2b) with the detachment and degradation of the dentin–resin interface (Figs 2c, 2d, 2e, 2f).

**Table 1:** Mean ± SD dye penetration distance (mm) at the tooth-resin margin/interface and the number of microleakage restorations prepared in vitro using MMA-TBB resin

Groups (n = 10)	Dye penetration distance (numbers of microleakage restorations)
Enamel	Cementum/dentin
1-1 etched 10 s	0 (0)	0 (0)
1-1 etched 30 s	0 (0)	0 (0)
Phosphoric etched 10 s	0 (0)	1.787 ± 0.485 (10)
Phosphoric etched 30 s	0 (0)	2.019 ± 1.292 (10)
0 = No dye penetration. Groups with dye penetration distances are not significantly different

The 1-1 etched 10 s and 30 s groups, with no dye penetration and a consistent hybrid layer, were further investigated in the oral (*in-vivo* study).

### In-vivo Study

**Fig 3:**
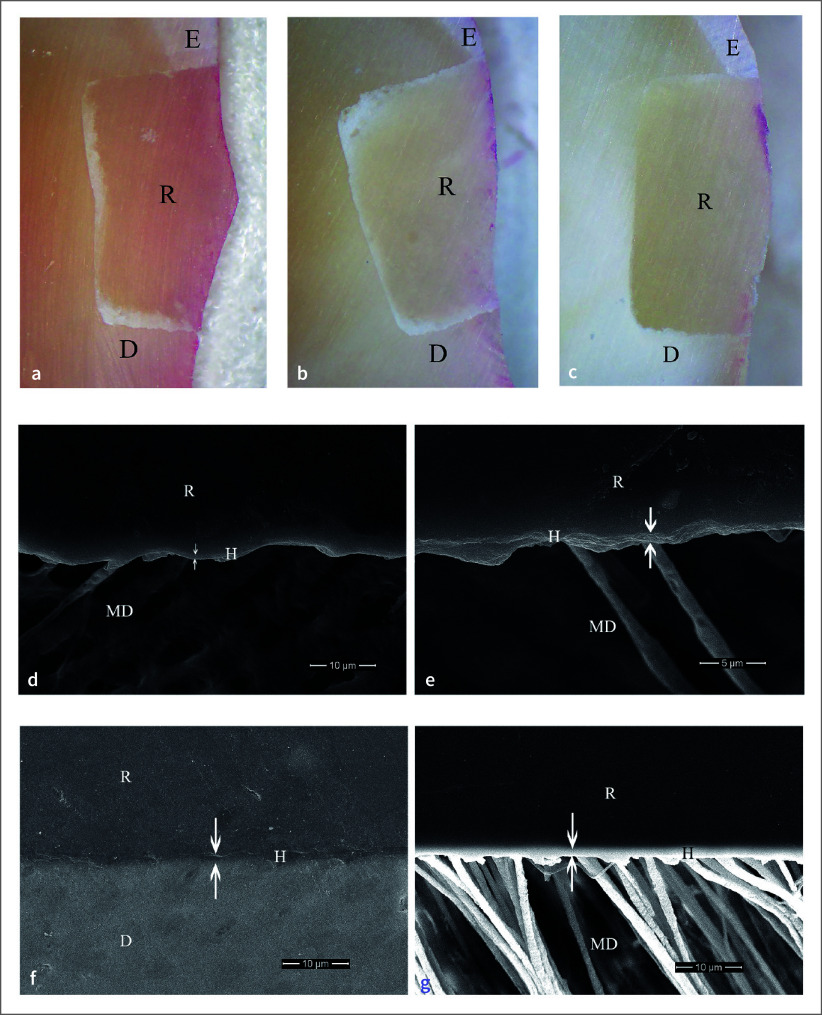
No dye penetration at tooth-restoration interface of the intraoral 4-META/MMA-TBB resin with 1-1 conditioning time of 10 s (a), 30 s (b), and 60 s (c) (original ×25, E = enamel, D = dentin, R = restoration). The 1–3 µm consistent hybrid layer after chemical challenge at dentin–resin interface: (d) 10 s, (e) 30 s, 60 s polished (f) and chemically challenged (g) specimens (original ×5000 (d, f, g), ×10,000 (e), D = dentin, MD = modified dentin, R = resin).

**Fig 4:**
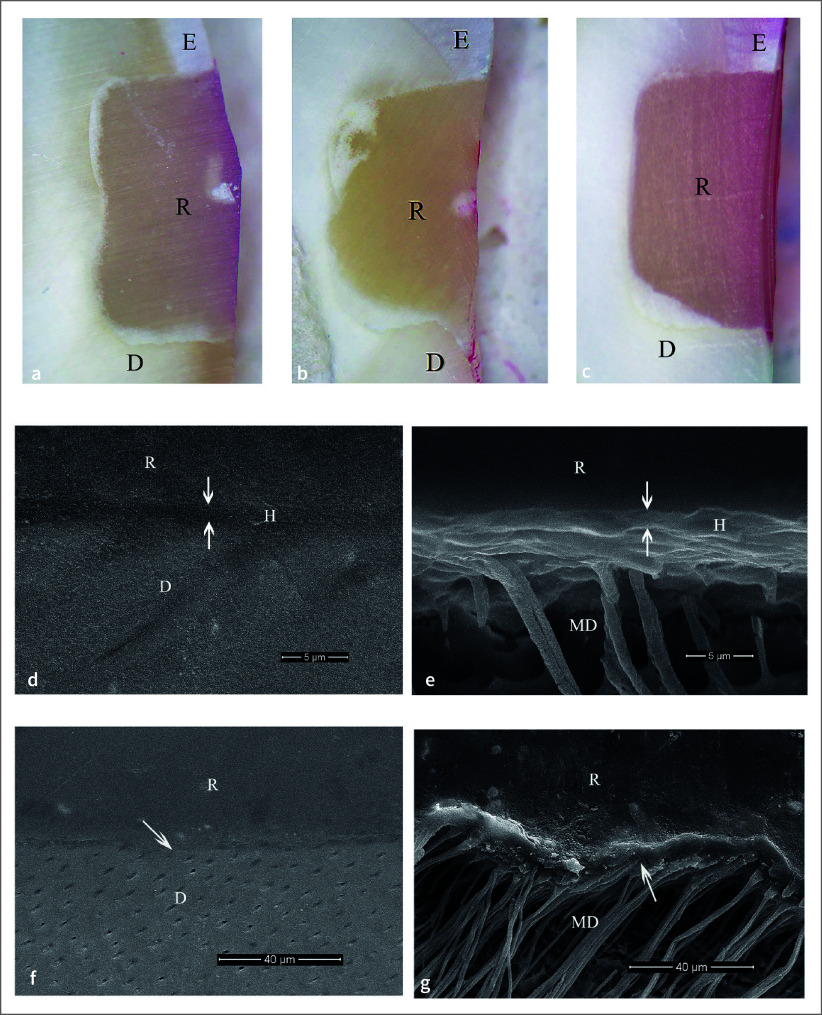
No dye penetration at tooth-restoration interface of the intraoral bonding using MMA-TBB resin with 1-1 conditioning time of 10 s (a), 30 s (b), and 60 s (c) (original ×25, E = enamel, D = dentin, R = restoration). The 2–3 µm consistent hybrid layer at dentin–resin interface in 30 s polished (d) and after chemical challenge (e) specimens, and the intact and continuous interface with a hybrid layer of 60 s polished (f) and chemically challenged (g) specimens (original ×10,000 (d, e), ×2000 (f, g), D = dentin, MD = modified dentin, R = resin).

**Fig 5:**
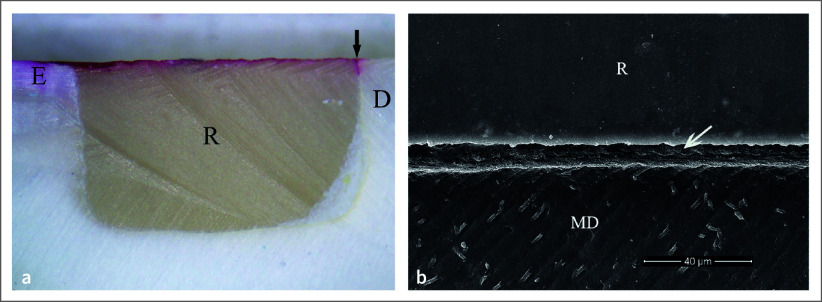
An intraoral specimen in 60 s 1-1 etching and MMA-TBB resin demonstrating: (a) leakage at cementum/dentin margin/interface (black arrow) (original ×25, E = enamel, D = dentin, R = restoration), (b) the detachment of the dentin–resin interface (white arrow) (original ×2000, MD = modified dentin, R = resin).

Four patients, three males and one female, with an average age of 54 years, were included. The distribution of restored teeth location and number of teeth is summarized in Table 2. No hypersensitivity, restoration defect, or detachment were found for all groups. All restorations had no dye penetration at the enamel margin/interface, while the dye penetration distance at the cementum/dentin margin/interface was found only in one specimen (Table 3). Two-way ANOVA and post-hoc least significant difference (LSD) test (α = 0.05) found no significantly different dye penetration distances between groups at the cementum/dentin margin/interface (p >0.05). For all etching periods of the 1-1 primerless wet bonding using 4-META/MMA-TBB resin adhesive, no leakage at the cementum/dentin margin/interface was found (Figs 3a, 3b, 3c) with a 1–3 µm consistent hybrid layer, which could resist the chemical challenge (Figs 3d, 3e, 3f, 3g). A leakage-free cementum/dentin margin/interface using MMA-TBB resin in all etching periods (Figs 4a, 4b, 4c) also provided a 1–3 µm stable hybrid layer after the chemical challenge (Figs 4d, 4e, 4f, 4g). One restoration with a 60 s conditioning period and MMA-TBB resin had a 0.13 mm leakage distance at the cementum/dentin-restoration interface (Fig 5a), of which the detachment of the dentin–resin interface was observed (Fig 5b).

**Table 2:** Distribution of tooth location and number of teeth for the dye penetration test


Tooth no.	16	15	14	13	12	11	21	22	23	24	36	35	34	33	32	31	41	43	45	46
No. of teeth	1	1	1	2	1	1	1	2	2	2	1	2	1	2	1	1	3	2	2	1


**Table 3:** Mean ± SD dye penetration distance (mm) at the tooth-restoration interfaces and number of microleakage restorations prepared intraorally

Groups (n =10)	Dye penetration distance (number of microleakage restorations)
4-META/MMA-TBB resin	MMA-TBB resin
Enamel	Cementum/dentin	Enamel	Cementum/dentin
1-1 etched 10 s	0(0)	0(0)	0(0)	0(0)
1-1 etched 30 s	0(0)	0(0)	0(0)	0(0)
1-1 etched 60 s	0(0)	0(0)	0(0)	0.013 ± 0.041 (1)
0 = No dye penetration. No significant difference was found for all groups.

## DISCUSSION

No dye penetration at the enamel and cementum/dentin margins and interfaces was recorded using 1-1 for 10 s and 30 s conditioning, primerless wet bonding without 4-META in MMA-TBB resin *in vitro* preparation (Fig 1) and intraoral (Fig 4) with the consistent hybridized dentin after immersion in HCl and NaOCl. The intraoral microleakage-free restorations were also found in 10 s, 30 s, and 60 s conditioning using 4-META/MMA-TBB with the consistent hybrid layer at the cementum/dentin–resin interface (Fig 3). The consistent hybrid layer before and after immersion in HCl and NaOCl of these groups *in vitro* (Figs 1c to 1f) and intraoral (Figs 3d to 3g, 4d to 4g) experiments suggests that using 1-1 conditioner, rinsed off, and blot-dried without primer on the enamel and dentin surface can provide a complete impregnation of both 4-META/MMA-TBB and MMA-TBB resin to form an impermeable hybrid layer that could resist the penetration of dye, HCl and NaOCl solutions. In contrast, detachment and degradation of the dentin–resin interface of 10 s and 30 s phosphoric acid conditioned groups (Figs 2c to 2f) confirms the existing demineralized dentin, where the dye penetrated through or the leakage pathway (Figs 2a, 2b) even in the more controllable laboratory preparation. These results strongly support the study hypothesis and have significant implications for the field of restorative dentistry.

Conditioning dentin with the less aggressive 1-1 conditioner for 10 to 60 s could completely remove the smear layer on prepared tooth surfaces and did not demineralize enamel or dentin too deeply, and created a thin hybrid layer (1–3 μm) using 4-META/MMA-TBB resin,^20,22,24^ compared with using 10-3 conditioning for 10 s (3–4 μm) with blot-drying^13^ or air-drying.^12,13,15,17,18,25^ The thinner permeable demineralized dentin allowed the complete impregnation of monomers to occur faster and easier. Therefore, either 4-META/MMA-TBB or MMA-TBB resin could create a 1–3 µm impermeable hybrid layer both in *in-vitro* and *in-vivo* restorations. In this hybrid layer coupled with the bulk-filled light-cured composite resin, no dye penetration was observed at the restorations’ margins/interfaces which suggests the stronger bond strength of these hybridized substrates compared to that of light-cured polymerization contraction.

The wet or moist bonding technique, in which the conditioned tooth surface is left moist to preserve channels between the collagen fibers for monomer penetration and impregnation, usually requires the primer to evaporate the remaining water for reliable bonding. Restorations using phosphoric acid for total etching, kept moist, primer applied, and bonded with resin adhesive could have severe leakage at the cementum/dentin margin/interface toward the pulp chamber^23^ and become detached in the short term.^9^ Cations such as Fe^3+^ or Ca^2+^ dissolved in acid are easily attracted by polyelectrolytes (PG, GAGs, PP) distributed along the collagen fibrils of demineralized dentin making them insoluble in the demineralized dentin, improving the permeability and dehydration rates of the demineralized dentin.^19^ The 10 s to 60 s 1-1 demineralized and blot-dried dentin had a high permeability, allowing water to totally evaporate and complete monomer impregnation, which can eliminate the priming step^22,24^ and the need for 4-META monomer promotor in the bonding adhesive in the laboratory and clinical treatment restorations as shown in this study. In contrast, the hybrid layer prepared in 1-1 conditioned dentin, air-dried, with or without 4-META in acetone primer, and 4-META/MMA-TBB resin application had less resin content compared with those with blot-dried using a primerless wet technique.^24^

The 4-META was introduced to adhesive dentistry as an excellent adhesion-promoting monomer to increase the rate of penetrability or diffusivity of co-monomers.^11^ It has been widely recognized as the component in bonding agents or luting adhesive for dentin bonding because of its hydrophilicity compatible with dentinal fluid. All restorations using 4-META/MMA-TBB adhesives had leakage-free margins and interfaces; this suggests the high reliability of using this bonding technique for vital teeth to protect dentin and pulp from external stimuli in the oral cavity. The average tensile bond strength using mini-dumbbell-shaped specimens test of 1-1 conditioning for 10 s to 60 s, primerless wet technique, and 4-META/MMA-TBB resin bonded to enamel and dentin *in vitro* was 20 MPa without adhesive failure at the tooth-resin interface, the same as using 10 s 10-3 conditioning and air-dried (Super-Bond C&B).^22^ Complete hybridization of resin into 1-1 etched dentin leaves no remaining demineralized dentin, results in an impermeable hybrid layer without dye penetration, and also provides a high tensile bond strength of 35–40 MPa when tested using direct tensile testing of mini-dumbbell-shaped specimens.^24^

The 1–3 µm 1-1 demineralized dentin, blot-dried and bonded with MMA-TBB, could provide an impermeable hybrid layer without dye penetration in the vital teeth, suggesting that the entire monomer impregnation and self-polymerized initiation occurred faster than the convection outward flow of dentinal fluid generated by pulpal tissue pressure. Fe^3+^ in the 1-1 conditioner and blot-dried technique is essential for MMA-TBB resin to obtain reliable dentin bonding in vital teeth without any signs or symptoms. Nearly all restorations (98%) in the MMA-TBB group were microleakage-free, suggesting high reliability when used in clinical treatment. Only one specimen with 60 s etching leaked at cementum/dentin margin/interface of 0.13 mm dye penetration distance (Fig 5a) and detachment of the dentin–resin interface (Fig 5b). The reason might be that water remained within the demineralized substrate before monomer application in that area and interfered with the complete impregnation of MMA-TBB. Thus, the remaining demineralized substrate was left as the leakage pathway, which may lead to postoperative hypersensitivity, marginal staining, or secondary caries. Therefore, technical caution by operators in visualizing the blot-dried surface is always needed clinically to achieve the best result. However, no postoperative hypersensitivity and defective margins were found clinically from this restoration as this leakage distance was too small.

A 10 s to 30 s 1-1 conditioning period is recommended for the intraoral preparation as it is enough time to create an impermeable hybrid layer without dye penetration, high tensile bond strength,^22^ and fewer resin tags in dentinal tubules (Figs 3d, 3e, 4e) compared with the 60 s conditioning (Figs 3g, 4g). In addition, the 60 s 1-1 dentin conditioning could remove more smear plugs, leading to the elimination of more dentinal fluid flow in demineralized dentin and more monomer diffusion into the dental pulp.

This study demonstrated that both 4-META/MMA-TBB resin and MMA-TBB resin had a high ability to integrate with the light-cured composite resin, as no dye penetration was found at their interface as shown under the low magnification microscope in this study. The MMA with slowly polymerized initiated TBB can diffuse deeper into the composite resin and copolymerize with fillers with unpolymerized- pendant-methacrylate groups in the Metafil composite resin. Thus, covalent bonding between MMA resin and composite resin possibly occurs.^15^ The MMA-based resin coupled with composite resin can resist the shear stress without detachment during the thin sectioning for SEM in this study and the ultra-thin sectioning for TEM examination (prepared without epoxy embedding).^25^

The novelty of this study is that 4-META/MMA-TBB resin or MMA-TBB resin bonded to the enamel and dentin conditioned with 1-1 for 10–60 s, rinsed, and blot-drying to remove smears and water provides a microleakage-free hybrid layer in the vital tooth. Therefore, restorations coupled with this hybrid layer can prevent oral stimuli, microorganisms, fluids, or acids from invading the dental pulp through a leakage pathway. Thus, patients do not have to worry about postoperative hypersensitivity, secondary caries, restoration detachment, and pulpal pathology after dental restoration. Further clinical trials are needed to prove long-term retention and functional load resistance on the bond strength of the 1-1 complete hybrid layer using MMA-TBB resin with/without 4-META.

## CONCLUSION

The MMA-TBB or 4-META/MMA-TBB resin bonded to the vital tooth cavity conditioned with 1-1 for 10 s to 60 s using a primerless wet technique and bulk-filling with light-cured composite resin could form leak-free restorations (no dye penetration at both enamel and cementum/dentin margins/interfaces).

### Conflict of Interest

The authors declare no conflicts of interest.

### Funding

This research did not receive any specific grant from funding agencies in the public, commercial, or not-for-profit sectors.

### Acknowledgments

The authors would like to thank Dr. John Harcourt, former Reader and Associate Professor, The University of Melbourne, for assistance with English language editing.

## References

[ref1] Alani AH, Toh CG (1997). Detection of microleakage around dental restorations: a review. Oper Dent.

[ref2] Brännström M (1966). Sensitivity of dentin. Oral Surg.

[ref3] Briso ALF, Mestrener SR, Delício G, Sundfeld RH, Bedran-Russo AK, De Alexandre RS, Ambrosano GMB (2007). Clinical assessment of postoperative sensitivity in posterior composite restorations. Oper Dent.

[ref5] Hanasaki Y, Watanabe A, Nakabayashi N (1999). The effect of water-soluble noncollagenous proteins on resin bonding to dentin. J Jpn Dent Mater.

[ref6] Hashimoto M, Ohno H, Kaga M, Endo K, Sano H, Oguchi H (2000). In vivo degradation of resin-dentin bonds in humans over 1 to 3 years. J Dent Res.

[ref7] Iwasaki Y, Toida T, Nakabayashi N (2004). Improved wet bonding of methyl methacrylate-tri-n-butylborane resin to dentin etched with ten percent phosphoric acid in the presence of ferric ions. J Biomed Mater Res A.

[ref8] Kidd EA (1976). Microleakage: a review. J Dent.

[ref10] Michael GB (1955). A Simple method of increasing the adhesion of acrylic filling materials to enamel surfaces. J Dent Res.

[ref12] Nakabayashi N, Ashizawa M, Nakamura M (1992). Identification of a resin-dentin hybrid layer in vital human dentin created in vivo: durable bonding to vital dentin. Quintessence Int.

[ref14] Nakabayashi N, Kojima K, Masuhara E (1982). The promotion of adhesion by the infiltration of monomers into tooth substrates. J Biomed Mater Res.

[ref16] Piemjai M, Adunphichet N (2022). Impact of hybrid layer formation on the 15-year survival, complications and failures of full-coverage retainers. J Prosthodont Res.

[ref17] Piemjai M, Chantarawej P, Nakabayashi N (2020). Evaluation of caries-free restorations bonded with various adhesive systems: in vitro study. Int J Dent.

[ref18] Piemjai M, Chantarawej P, Nakabayashi N, Garcia-Godoy F (2017). Prognosis test by visualization of demineralized dentin under restorations to prevent initial wall-lesions initiated by lactic acid. Am J Dent.

[ref19] Piemjai M, Iwasaki Y, Nakabayashi N (2003). Influence of dentinal polyelectrolytes on wet demineralized dentin, a bonding substrate. J Biomed Mater Res A.

[ref20] Piemjai M, Nakabayashi N (2001). Effect of dentin conditioners on wet bonding of 4-META/MMA-TBB resin. J Adhes Dent.

[ref21] Piemjai M, Thaveeratana A, Nakabayashi N (2010). Marginal integrity between a prefabricated composite block and enamel, DEJ, and dentin. Am J Dent.

[ref22] Piemjai M, Waleepitackdej O, Garcia-Godoy F (2023). Marginal micro-seal and tensile bond strength of a biopolymer hybrid layer coupled with dental prosthesis using a primerless-wet system. Polymers.

[ref23] Piemjai M, Waleepitackdej O, Garcia-Godoy F, Nakabayashi N (2011). Dentin protection by a primer-less adhesive technique. Am J Dent.

